# Personalized Informational Support for Patients With Hypertension: Single-Arm Pretest-Posttest Study

**DOI:** 10.2196/82147

**Published:** 2026-01-26

**Authors:** Chuanying Huang, Rong Yang, Lidi Liu, Yu Jia, Xiaoyang Liao

**Affiliations:** 1 Department of Emergency Medicine, West China Hospital of Sichuan University, Chengdu, Sichuan, China; 2 General Practice Ward/International Medical Center Ward, General Practice Medical Center, West China Hospital of Sichuan University, No. 37 Guoxue Alley, Wuhou District, Chengdu, Sichuan, 610041, China, +86 18080085361

**Keywords:** hypertension, adherence, blood pressure, social support, informational support

## Abstract

**Background:**

Informational support has been demonstrated to enhance patients’ treatment adherence. However, which specific mode of informational support is more effective for patients with hypertension remains undetermined.

**Objective:**

The primary objective of this study was to conduct a feasibility exploration of personalized informational support in patients with hypertension using a single-arm pretest-posttest design.

**Methods:**

A prospective, single-center, pretest-posttest study was used to investigate the feasibility of providing an informational support intervention to patients with hypertension attending a community health facility in Chengdu, China. The intervention combined in-person follow-ups and telephone counseling. Adherence and clinical outcomes (blood pressure, ambulatory blood pressure, and laboratory tests) were measured at baseline and the postintervention time point. Patients’ health behaviors were assessed at baseline and the postintervention time point using validated structured questionnaires. Descriptive statistics and effect sizes were calculated to determine clinically important changes relative to baseline.

**Results:**

Significant improvements were observed: medication adherence scores increased by 0.65 points (95% CI 0.38-0.91; *P*<.001). Nutrition scores increased by 1.31 points (95% CI 0.53-2.09; *P*<.001), interpersonal relationship scores increased by 1.17 points (95% CI 1.03-2.02; *P*=.007), health responsibility scores increased by 2.42 points (95% CI 0.33-3.80; *P*=.001), and the total Health-Promoting Lifestyle Profile II–Revised score significantly increased by 6.81 points (95% CI 3.01-10.61; *P*=.001). Nighttime systolic blood pressure decreased significantly by 5.07 mm Hg (95% CI −8.12 to –2.01; *P*=.001), and nighttime diastolic blood pressure decreased significantly by 3.39 mm Hg (95% CI −5.12 to −1.67; *P*<.001).

**Conclusions:**

This feasibility study found that a structured informational support intervention was well accepted (93/100, 93% retention) and was associated with preliminary improvements in medication adherence and nocturnal blood pressure. These findings suggest potential benefits and support the need for a definitive randomized controlled trial to establish efficacy.

## Introduction

### Background

Hypertension is a major risk factor for many diseases. World Health Organization data show that, worldwide, 33% of people aged 30 to 79 years have hypertension, constituting 1.3 billion people. In China, the prevalence is 27.9% with 245 million patients, but only 41% are aware, 34.9% are treated, and 11% have controlled blood pressure [1]. Poor treatment adherence is the main reason for uncontrolled blood pressure. The World Health Organization defines treatment adherence as following the physician’s plan, including medication and lifestyle. Nonadherence makes it 2.15 times harder to control blood pressure, 2.08 times more likely to have complications, and 1.38 times more likely to die [2]. Thus, improving hypertension management is urgent and important.

Despite significant advances in medication accessibility, treatment adherence remains suboptimal. This nonadherence crisis necessitates a paradigm shift toward nonpharmacological determinants of treatment outcomes. Informational support is an important part of social support, defined as the strategic delivery of evidence-based guidance, therapeutic counseling, and health literacy interventions aimed at stress mitigation and illness coping skill development [3]. This knowledge translation process empowers patients to navigate complex treatment regimens while optimizing self-efficacy in chronic disease management, simultaneously alleviating patients’ anxiety and perceived uncertainty to enhance therapeutic adherence.

There is emerging evidence suggesting that nontailored informational support modalities may engender maladaptive health beliefs, undermining medication adherence through distorted risk-benefit perceptions [4] and, ultimately, compromising blood pressure trajectory stabilization [5]. A recent study published in the *Journal of the American Medical Association* demonstrated that SMS text message reminders for patients who delayed initiating cardiovascular medication supplementation did not enhance medication adherence or decrease clinical events at the 12-month follow-up [6]. Notwithstanding these gaps in delivery optimization, systematic reviews highlight promising yet inconsistent efficacy signs for dyadic communication strategies, particularly protocol-driven face-to-face follow-up (mean difference 1.00, 95% CI 0.46-1.54) [7] vs structured telephone consultations (risk ratio=1.30, 95% CI 1.07-1.59) [8].

However, the implementation pathways of informational support in hypertension management remain underexplored. Current clinical practices face several challenges: (1) fragmented health education models that lack systematic integration and refinement; (2) the provision of standardized informational support failing to account for patient individuality, resulting in inadequate attention to critical needs; and (3) overreliance on clinic-based in-person sessions. To address these gaps, this study developed a hierarchical progressive informational support framework. The integrated intervention synergizes personalized in-person follow-up (for trust building and rapport establishment) with telephone-delivered counseling, forming a dual-modality framework that has demonstrated potential efficacy in prior feasibility studies [9]. Therefore, we conducted this prospective single-arm study to assess the acceptability of the personalized informational support intervention and preliminary changes in clinical indicators to inform a future randomized controlled trial (RCT).

### Objectives

This study used a single-arm pretest-posttest design to explore the feasibility of personalized informational support for patients with hypertension. The primary objectives were to (1) assess the implementation feasibility of the intervention, (2) observe preliminary changes in therapeutic adherence and blood pressure control following the intervention, and (3) inform the design and sample size calculation for a future RCT.

## Methods

### Study Design and Participants

A prospective, single-center, pretest-posttest study was conducted at the Cuqiao Community Health Service Center in Wuhou District, Chengdu. This study adopted a consecutive enrollment method targeting patients with hypertension visiting community clinics, with enrollment stopped after 100 participants were included. Recruitment began on May 25, 2019, and ended on June 15, 2019. This study adhered to the reporting content of the Transparent Reporting of Evaluations With Nonrandomized Designs guidelines.

The diagnostic criteria for hypertension were established as follows: (1) office-measured systolic blood pressure (SBP) of ≥140 mm Hg and/or diastolic blood pressure (DBP) of ≥90 mm Hg obtained through 3 separate measurements on different days and (2) documented history of hypertension with current use of antihypertensive medications, with confirmed diagnosis maintained even when SBP <140 mm Hg and/or DBP <90 mm Hg.

The inclusion and exclusion criteria are shown in Textbox 1.

Textbox 1. **Inclusion and exclusion criteria.**

**Inclusion criteria**

- Essential hypertension
- Residence in the community for >1 year
- Age range of 40 to 80 years

**Exclusion criteria**

- Hypertensive emergency
- Acute-phase myocardial infarction
- Acute-phase cerebral infarction
- Pregnancy or lactation
- Inability to participate in study procedures due to significant cognitive impairment, hearing deficits, or physical disabilities

### Sample Size Calculation

In previous studies, an educational intervention by health care professionals was shown to reduce SBP by 8 mm Hg with a paired-difference SD of 22.86 mm Hg [10]. Using an α value of .05 (2 sided) and 80% power, a minimum of 65 participants was required. Accounting for 20% attrition, we planned to recruit 92 participants. A total of 100 people were recruited in the end.

### Intervention

#### Overview

The current hypertension management model in communities primarily relies on clinic-based management, with a greater emphasis on medication adjustment. There is a significant lack of attention to informational support, and the health education provided is often sporadic and superficial, lacking systematic and personalized approaches. In this study, we designed a personalized informational support strategy that integrates the educational level and age of patients to formulate the support tactics. Moreover, the emphasis of the support is dynamically adjusted according to the patients’ comprehension and mastery levels.

The informational support intervention comprised monthly 30-minute face-to-face sessions delivered by trained community physicians over 3 months. Content included knowledge about hypertension, lifestyle guidance, medication adherence strategies, and monitoring and follow-up protocols. All sessions were standardized using a structured manual, and physicians received uniform preimplementation training.

Additionally, uniformly trained community nurses provided monthly telephone counseling interventions (approximately 10‐15 minutes per call). These calls focused on reviewing home blood pressure measurements from the previous month, assessing dietary habits and physical activity levels, and evaluating medication adherence. Nurses also encouraged patients to follow the lifestyle recommendations in the health handbook distributed to each patient before the start of the study, addressed patients’ concerns, and reminded them of upcoming follow-up appointments.

#### In-Person Follow-Up

At the initial consultation, patients’ age and highest educational attainment were recorded through a structured questionnaire, and health education materials were distributed covering hypertension risks; lifestyle recommendations (dietary guidelines and physical activity regimens); quantitative self-assessment methods; and management of hypertension-related conditions, including blood glucose and lipid control. During the 3-month intervention, patients received monthly 30-minute follow-up sessions. The content of these sessions was tailored to the patients’ educational level, health literacy, and age to optimize the delivery and comprehension of informational support.

#### Tailoring by Educational Level and Health Literacy

For individuals with lower educational attainment or limited health literacy, information was delivered using low-literacy principles. This involved the following:

- Simplified language: avoiding complex medical terminology (eg, “hypertension” was consistently referred to as “high blood pressure”). Instructions were concrete and action-oriented (eg, “Take this pill every morning after breakfast” instead of “Administer the antihypertensive agent upon waking”).
- Visual-centric modalities: heavy reliance on pictograms, color-coded charts, and illustrative diagrams. For instance, medication schedules were presented using images of the sun and moon to denote day and night doses, and dietary recommendations used visual portion size guides (eg, a clenched fist representing 1 serving of vegetables).
- Interactive demonstration: instead of passive receipt of information, patients were actively involved in demonstrations, such as practicing with a blood pressure monitor or using a salt restriction spoon with mock food.

Conversely, for individuals with higher educational attainment, the information included more comprehensive explanations of the underlying pathophysiology and the evidence base for recommendations. Patients were actively engaged in shared decision-making regarding their therapeutic regimens, discussing the pros and cons of different lifestyle choices.

#### Tailoring by Age Group

For middle-aged patients (41-60 years), a hybrid approach was used. They received access to a mobile health app alongside printed materials, allowing them to choose their preferred information channel.

**Figure 1.**
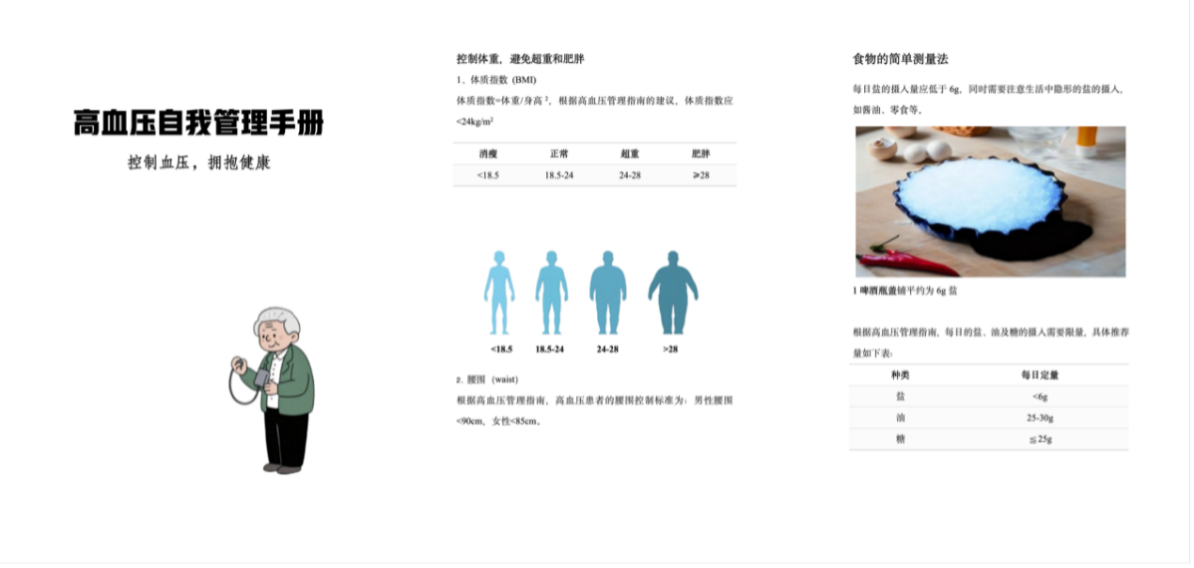
Health education materials distributed to patients with hypertension in the Cuqiao community before the intervention.

For older patients (>60 years), who might be less comfortable with digital technology, information was delivered primarily through face-to-face interactions and enlarged-print materials. The content was paced more slowly, with key points repeated for emphasis. The health education materials are shown in Figure 1.

#### Training for General Practitioners and Intervention Framework

General practitioners received structured competency training via the Community Cardiovascular Disease Prevention and Management Platform developed by the National Center for Cardiovascular Diseases. Key objectives included (1) establishing an evidence-based knowledge repository for hypertension and coronary heart disease management, (2) enabling real-time specialist teleconsultations, and (3) implementing an efficient clinical decision support question-and-answer system. These interventions targeted measurable improvements in primary care physicians’ diagnostic accuracy and guideline-adherent therapeutic decision-making within the trial cohort.

During the first follow-up visit, all patients received an approximately 30-minute standardized informational support intervention, which included disease knowledge education, lifestyle guidance, treatment plan discussions, and follow-up monitoring instructions, among other components. A self-designed quiz was administered before each of the 2 subsequent follow-up visits to assess the patients’ understanding of the informational support content from the previous session. The quiz consisted of a total of 9 questions, with 3 questions for each dimension. On the basis of the quiz results, targeted support was provided to address the patients’ weak areas to consolidate the effectiveness of the informational support. The detailed intervention framework is shown in Table 1.

**Table 1.** In-person follow-up protocol for hypertension management: a structured face-to-face intervention framework targeting blood pressure control and lifestyle modification.

Follow-up content and intervention module	Standardized intervention content
Disease knowledge	
Disease education	Pathophysiology: hypertension staging and target organ damage warning signs (heart, brain, kidney, or retina) Metabolic targets (BP^a^: <140/90 mm Hg at the clinic and <135/85 mm Hg at home; fasting glucose: ADA^b^ standard of <5.6 mmol/L; LDL-C^c^: ACC^d^ and AHA^e^ guideline of <2.6 mmol/L)
Lifestyle recommendations	
Nutrition management	Sodium restriction: salt restriction spoon (≤6 g per d) and identification of hidden sodium sources Dietary modification: 300-500 g of vegetables (>50% dark colored), 200-350 g of fruits (low GI^f^ preferred), and >50% of whole grains
Addiction cessation	Stepwise smoking cessation: nicotine replacement therapy options and withdrawal symptom management Alcohol control: alcohol abstinence
Exercise prescription	Personalized exercise plan: target heart rate zone (Karvonen formula); 3-5 sessions of 40-60 min per wk of aerobic exercise and 2 sessions per wk of resistance training Sedentary behavior intervention: 2-min activity breaks every 30 min
Weight management	Caloric deficit protocol: 300-500 kcal per d deficit and 1.2-1.5 g per kilogram of protein intake (normal renal function) Central obesity control: waist circumference monitoring (<90 cm for male individuals and <85 cm for female individuals)
Treatment plan	
Medication	Conduct patient-centered therapeutic education: explain pharmacological mechanisms, potential adverse effects, and medication administration protocols; collaboratively identify adherence barriers; and codevelop personalized strategies to overcome these obstacles
Monitoring and follow-up	
Monitor and document BP readings and tailor subsequent follow-up care	Instruct the patient on how to monitor and record BP in a standardized manner and provide guidance on the timing of the next follow-up visit

a BP: blood pressure.

b ADA: American Diabetes Association.

c LDL-C: low-density lipoprotein cholesterol.

d ACC: American College of Cardiology.

e AHA: American Heart Association.

f GI: glycemic index.

#### Telephone Follow-Up

Monthly telephone consultations were conducted by trained community nurses. If the initial call went unanswered, up to 3 additional attempts were made on separate days within the same month. Each consultation lasted approximately 10 to 15 minutes. During these calls, nurses (1) assessed patients’ home blood pressure measurements, dietary habits, and physical activity levels over the preceding month; (2) evaluated medication adherence using the scale recommended by the 2018 Chinese hypertension guidelines; (3) provided tailored encouragement for adhering to recommended lifestyle modifications; (4) addressed patient concerns; and (5) reminded patients to attend their next scheduled in-person follow-up visit.

### Outcome

This study explicitly took medication adherence as the primary outcome, with blood pressure, lifestyle adherence, and other blood test indicators as exploratory outcomes.

#### Primary Outcome

Patient medication adherence was measured via a scale recommended by the 2018 Chinese hypertension guidelines [10] at baseline and 3 months. This scale demonstrated good reliability and validity, with a Cronbach α coefficient of 0.736 and a content validity index of 0.970. On the basis of the total score, medication adherence was categorized into 3 levels: low (<6 points), moderate (≥6 and <8 points), and high (8 points; Table S1 in Multimedia Appendix 1).

#### Secondary Outcome

Lifestyle adherence was evaluated using the healthy lifestyle index (HLI), with total scores ranging from 0 to 9. Higher scores indicate superior adherence behaviors. The HLI comprises 9 risk factors, specifically including smoking (0‐2 points), BMI (0‐1.5 points), physical activity (0‐1.5 points), high-sodium diet (0‐1 point), alcohol consumption (0‐1 point), low fruit intake (0‐0.5 points), sleep (0‐0.5 points), whole grain intake (0‐0.5 points), and sugar-sweetened beverage intake (0‐0.5 points). This proportion allocation is based on the ranking of disease burdens (measured using disability-adjusted life year percentages) of various risk factors in the 2017 Global Burden of Disease Study [11]. Following the principle of “higher risk, higher weight; lower risk, lower weight,” scores are assigned to 9 key lifestyle dimensions, with a total of 9 points. This design aims to scientifically reflect the differential health impacts of each behavior and achieve quantitative assessment of lifestyle adherence. Specific items and determination criteria are shown in Table S2 in Multimedia Appendix 1. The reliability of the HLI was tested in 50 pilot participants: internal consistency was acceptable (Cronbach α=0.71), and test-retest reliability over 2 weeks was good (intraclass correlation coefficient=0.82).

Blood pressure metrics comprised clinic measurements and 24-hour ambulatory monitoring. For clinic blood pressure measurement, the Omron HBP-1300 was used, which has passed the British Hypertension Society A/A validation protocol and the European Society of Hypertension International Protocol 2010 revision. For ambulatory blood pressure monitoring, the Omron M2-180A was used; this model fulfills the Association for the Advancement of Medical Instrumentation and International Organization for Standardization 81060-2:2018 accuracy criteria. There should be no fewer than 20 active readings during the daytime and no fewer than 7 active readings during the nighttime. The total number of active readings within a 24-hour period must account for more than 70% of all measured instances. Health-promoting lifestyle was assessed using the Health-Promoting Lifestyle Profile II–Revised, scored on a scale from 1 (“never”) to 4 (“always”), and was classified into 6 factors: interpersonal relationships, health responsibility, stress management, nutrition, physical activity, and spiritual growth. The higher the score, the better the health-promoting lifestyle. It was specifically tailored for the Chinese population by Cao et al [12]. Reliability analyses have indicated acceptable internal consistency (Cronbach α=0.63‐0.81), moderate split-half reliability (0.64‐0.78) for all subscales, and test-retest stability (*r*=0.69). Height and weight were assessed using a flexible measuring tape and a body composition analyzer (V-body HBF-371). BMI was calculated using the formula BMI = weight (kg)/height (m) squared. Waist circumference was also measured using flexible measuring tape. Fasting blood samples (3-5 mL) were collected from the median cubital vein during morning hours. Samples were processed within 30 minutes after collection through centrifugation at 3000 revolutions per minute for 10 minutes at 4 °C.

#### Covariates

This study included age, sex, marital status, educational level, living situation, and household size as covariates.

### Data Analysis

#### Overview

Analyses were conducted on outcomes at baseline and the 3-month postintervention follow-up. Data entry was performed independently by 2 researchers using EpiData (version 3.1; EpiData Association), with subsequent statistical processing completed in SPSS (version 26.0; IBM Corp). For quantitative analysis, univariate descriptive statistics and frequency distribution calculations were performed on the complete sample. Differences in mean values before and after the intervention were evaluated using paired *t* tests (2 tailed). Effect size, a method quantifying therapeutic impact, was used to interpret intervention efficacy: 0.2 indicates a small effect, 0.5 indicates a medium effect, and 0.8 indicates a large effect. In addition to demonstrating clinical relevance through effect sizes, statistically significant differences were observed in primary outcome measures (*P*<.05).

#### Handling of Missing Data

Missing data were managed using a 2-pronged approach to ensure the robustness of our findings. The primary issue involved missing postintervention outcome measures, which occurred in 7% (7/100) of the participants, primarily due to loss to follow-up.

#### Complete Case Analysis

As an initial sensitivity analysis, we included only participants with complete data at both baseline and the 3-month follow-up (n=93). This approach served to assess whether the results were sensitive to the exclusion of participants with missing data.

#### Multiple Imputation

For the primary analysis, we used multiple imputation by chained equations using the *mice* package (version 3.14.0) in R (R Foundation for Statistical Computing) to handle missing data. This method was chosen as it is a gold standard for longitudinal data, preserves statistical power, and reduces bias under the missing at random assumption. The imputation model included all baseline variables (eg, age, gender, and baseline blood pressure measures), intervention exposure indicators, and all outcome measures. We generated 10 imputed datasets, and the results were pooled according to the Rubin rules to obtain final estimates and SEs that accounted for the uncertainty inherent in the imputation process.

The complete case analysis provided a benchmark for assessing potential bias introduced by complete case exclusion. The multiple imputation approach, being more robust, was used for the primary inference. The consistency of the intervention effects (statistically significant improvements in primary outcomes) across both analytical approaches enhanced our confidence in the stability and validity of the study conclusions.

### Ethical Considerations

This single-center, pretest-posttest study involving human participants was reviewed and approved by the institutional review board (IRB) of the West China Hospital, Sichuan University (registration 2019; review 573). The IRB specifically evaluated the study design (self–pretest-posttest comparison) to ensure minimal risk to participants, focusing on the safety of the health education intervention and the confidentiality of personal health data.

Written informed consent was obtained from all participants before the baseline assessment. The consent form clearly explained (1) the study purpose (to evaluate the impact of a health behavior intervention on clinical and behavioral outcomes via self-comparison), (2) the procedures (baseline data collection; 3-month intervention, including health education manuals and dietary counseling; and postintervention follow-up), (3) the potential risks (eg, no improvement in outcomes and temporary inconvenience from data collection), and (4) participants’ rights (to withdraw at any time without affecting clinical care and access to their deidentified data summary). For participants with low literacy, verbal consent was obtained in the presence of a study nurse (witnessed and documented with the witness’s signature), as approved by the IRB.

All personal identifiable information (eg, names and medical record numbers) was removed at the time of data entry. Deidentified datasets were used for all analyses, ensuring that participants’ privacy was protected throughout the study.

All participants in this study volunteered to take part and received no financial compensation.

This study was registered with the Chinese Clinical Trial Registry under the identifier ChiCTR2000039621.

## Results

### Patient Characteristics

**Figure 2.**
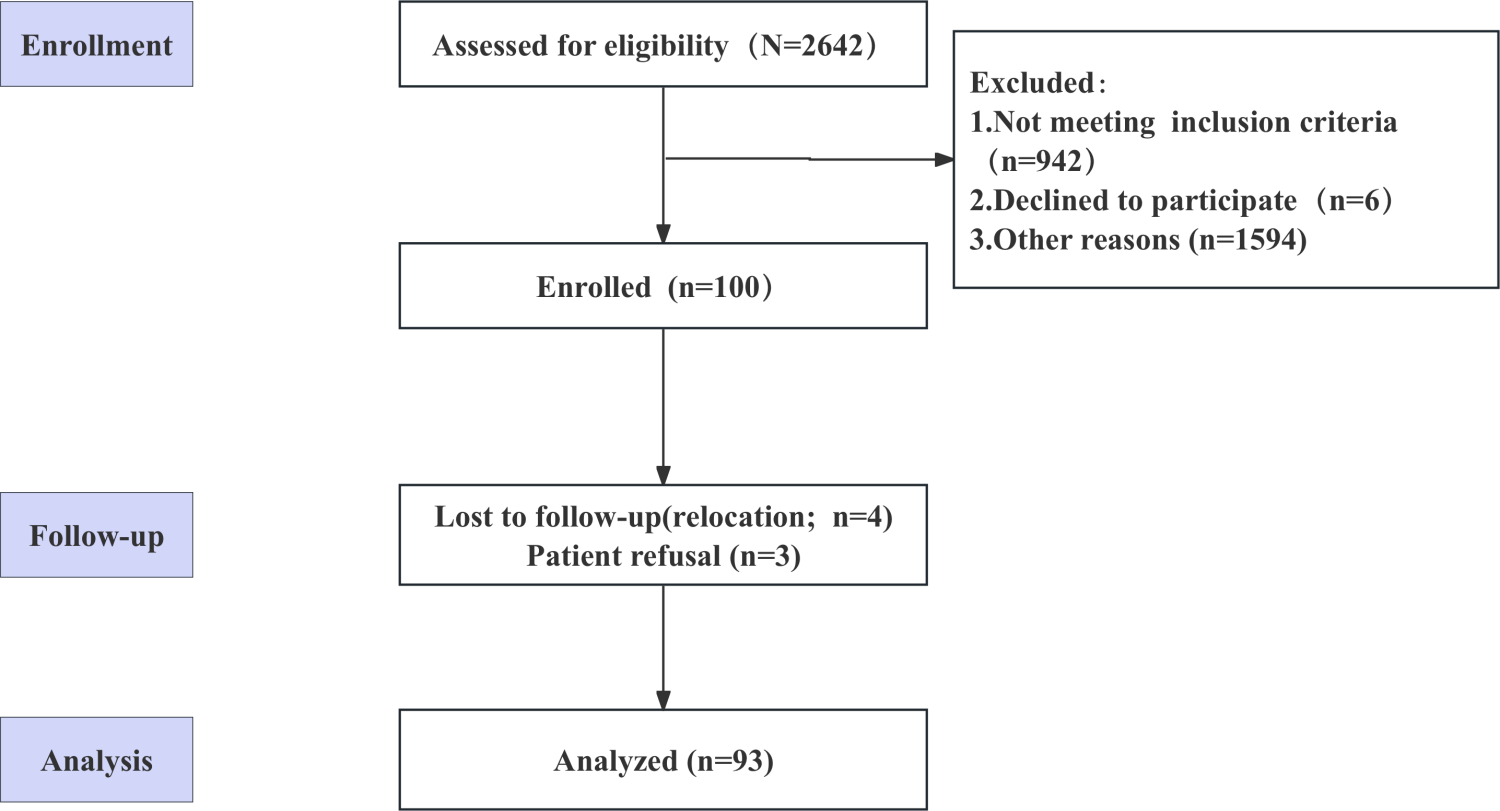
Participant flow diagram of a before-and-after study on an informational support intervention for patients with hypertension (postintervention time point and 3-month follow-up assessments; Cuqiao Community Health Service Center).

We enrolled 100 consecutive patients with hypertension from the general outpatient clinic of a community health care center between May 2019 and June 2019. All 100 patients completed the baseline assessment, 3 (3%) withdrew from the study, and 4 (4%) were lost to follow-up due to relocation. Hence, 93% (n=93) completed assessments at 3 months. Details are provided in Figure 2.

The mean age of the study participants was 60.88 (SD 10.43) years. Most were female (59/100, 59%) and married or cohabiting (96/100, 96%), had a high school education or lower (90/100, 90%), and were living with family (98/100, 98%). Details are provided in Table 2.

**Table 2.** Baseline characteristics of patients with hypertension at the Cuqiao Community Health Service Center (N=100).

Characteristic	Values
Age (years), mean (SD)	60.88 (10.43)
Sex, n (%)	
Male	41 (41)
Female	59 (59)
Marital status, n (%)	
Married or cohabiting	96 (96)
Divorced	0 (0)
Widowed	4 (4)
Educational level, n (%)	
Primary school and lower	29 (29)
Middle school	28 (28)
High school	33 (33)
College	8 (8)
University	1 (1)
Postgraduate	1 (1)
Living alone, n (%)	
No	98 (98)
Yes	2 (2)
Number of family members participants lived with, n (%)	
1-2	27 (27)
3-4	25 (25)
≥5	48 (48)

### Pre- and Postintervention Differences in Adherence

The medication adherence score exhibited a statistically significant improvement of 0.65 points (95% CI 0.38 to 0.91; *P*<.001) at 3 months after the intervention compared to baseline measurements. In contrast, the lifestyle adherence score exhibited a modest increase of 0.24 points (95% CI 0.17 to 0.64; *P*=.25) following the intervention.

### Health Promotion Behaviors

Statistically significant differences were observed in the nutrition (*P*<.001), interpersonal relationships (*P*=.001), health responsibility (*P*=.001), and overall health-promoting behavior scores (*P*=.001) of patients with hypertension before vs after the intervention. However, no statistically significant differences were detected in the physical activity (*P*=.54), stress management (*P*=.14), and spiritual growth (*P*=.17) dimensions. Details are provided in Table 3.

**Table 3.** The difference in health behaviors before and after an informational support intervention in a before-and-after study of patients with hypertension at the Cuqiao Community Health Service Center.

Domain	Baseline, mean (SD)	3 months, mean (SD)	Mean difference (95% CI)	*P* value	Cohen *d*
Nutrition (0-24)	11.17 (2.90)	12.48 (1.99)	1.31 (0.53 to 2.09)	<.001	0.353
Physical activity (0-32)	15.86 (4.35)	16.24 (4.10)	0.38 (–0.84 to 1.61)	.54	0.065
Stress management (0-20)	8.54 (3.32)	9.17 (2.77)	0.63 (–0.22 to 1.48)	.14	0.154
Interpersonal relationships (0-20)	9.57 (3.16)	10.76 (2.39)	1.17 (0.33 to 2.02)	.007	0.287
Health responsibility (0-44)	16.62 (4.62)	19.03 (5.55)	2.42 (1.03 to 3.80)	.001	0.364
Spiritual growth (0-20)	8.56 (3.48)	9.22 (3.09)	0.67 (–0.30 to 1.64)	.17	0.154
Total scores (0-160)	70.29 (12.72)	77.10 (13.53)	6.81 (3.01 to 10.61)	.001	0.379

### Clinical Outcomes

**Figure 3.**
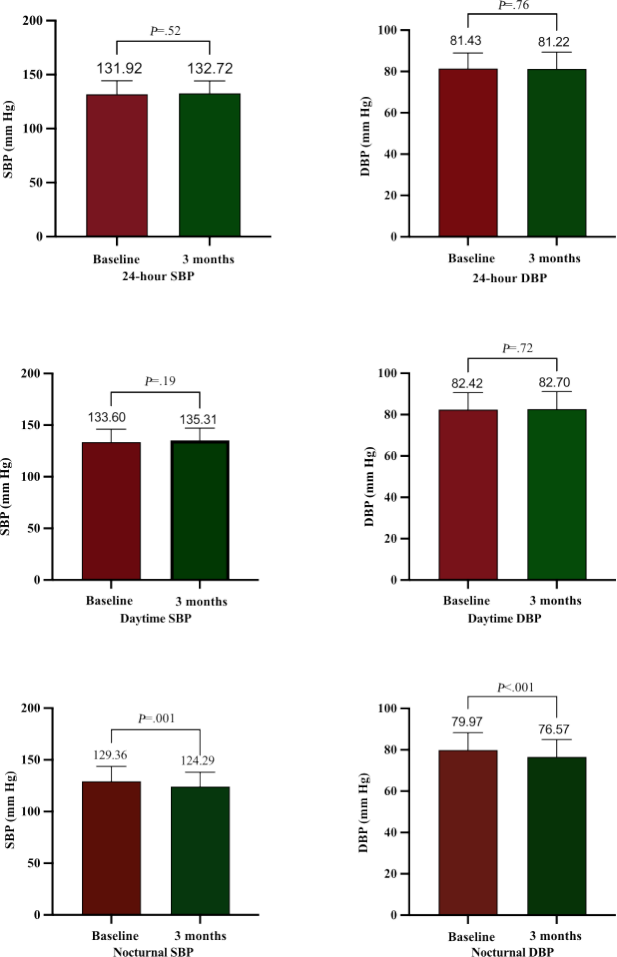
Impact of informational support on ambulatory blood pressure in patients with hypertension: comparison of baseline vs 3 months after the intervention in a before-and-after controlled study (Cuqiao Community Health Service Center). DBP: diastolic blood pressure; SBP: systolic blood pressure.

The postintervention nocturnal blood pressure parameters exhibited statistically significant reductions, with SBP decreasing by 5.07 mm Hg (95% CI –8.12 to –2.01; *P*=.001) and DBP decreasing by 3.39 mm Hg (95% CI −5.12 to −1.67; *P*<.001). In contrast, no statistically significant alterations were observed in daytime SBP or DBP (*P*=.19 and *P*=.72, respectively), 24-hour ambulatory blood pressure metrics (SBP: *P*=.52; DBP: *P*=.76), waist circumference (Δ=–0.08 cm; *P*=.89), or BMI (Δ=0.27 kg/m^2^; *P*=.17) following the intervention protocol. Office blood pressure measurements, BMI, waist circumference data, and laboratory indicators are presented in Table 4, whereas ambulatory blood pressure monitoring results are illustrated in Figure 3.

**Table 4.** The difference in clinical outcomes before and after an informational support intervention in a before-and-after study of patients with hypertension at the Cuqiao Community Health Service Center.

Variable	Baseline, mean (SD)	3 months, mean (SD)	Mean difference (95% CI)	*P* value	Cohen *d*
Systolic blood pressure (mm Hg)	131.80 (11.76)	131.82 (10.38)	0.07 (−2.37 to 2.51)	.96	0.006
Diastolic blood pressure (mm Hg)	78.17 (8.45)	77.87 (7.56)	−0.39 (−2.30 to 1.52)	.68	0.042
BMI (kg/m^2^)	25.17 (2.79)	25.44 (3.22)	0.27 (0.12 to 0.66)	.17	0.293
Waist circumference (cm)	86.07 (7.43)	85.99 (6.18)	−0.08 (−1.18 to 1.03)	.89	0.014
Glucose (mmol/L)	5.56 (1.26)	5.40 (0.75)	−0.17 (−0.45 to 0.11)	.24	0.124
Triglycerides (mmol/L)	1.61 (0.89)	1.56 (0.66)	−0.06 (−0.24 to 0.13)	.54	0.064
Total cholesterol (mmol/L)	5.00 (1.04)	4.86 (0.87)	−0.14 (−0.39 to 0.11)	.27	0.116
Low-density lipoprotein cholesterol (mmol/L)	1.58 (0.23)	1.56 (0.35)	−0.02 (−0.08 to 0.05)	.64	0.114
High-density lipoprotein cholesterol (mmol/L)	2.76 (0.86)	2.64 (0.97)	−0.11 (−0.31 to 0.09)	.28	0.049

## Discussion

This community-based implementation study addressed the aim stated in the Introduction section to evaluate whether a structured informational support intervention could improve adherence, lifestyle behaviors, and clinical outcomes in patients with hypertension. It provides novel evidence of feasibility (93/100, 93% retention, exceeding the National Institutes of Health’s 80% benchmark) and preliminary efficacy, with findings aligned with the reach, effectiveness, adoption, implementation, and maintenance framework’s reach and adoption dimensions, suggesting strong potential for scale-up in resource-limited settings.

### Adherence Indicators

According to social support theory, informational support enhances adherence by boosting health literacy (eg, helping patients understand how medications control blood pressure) and self-efficacy (eg, building confidence to manage side effects) [13]. Consistent with this, we observed clinically meaningful improvements in medication adherence scores. Notably, 73.1% (68/93) of the patients showed improved adherence, a rate comparable to that of recent RCTs of health literacy interventions [14]. While HLI scores increased nominally, this aligns with prior work showing that lifestyle modification is more challenging than medication adherence [15]. This may be attributed to the fact that the short-term benefits of changes in lifestyle are less pronounced than those of medications, which has also led patients to place less emphasis on long-term lifestyle modifications. This suggests that future research should pay greater attention to lifestyle changes, enhance the intensity of related interventions, extend intervention durations, and prioritize the monitoring of adherence to lifestyle interventions so as to achieve better intervention outcomes.

### Health Behavior

Traditional hypertension interventions often use one-off health education or target single groups, leading to low engagement [16]. The improvements in nutrition and interpersonal relationship scores in our study may be associated with the intervention. However, physical activity remained unchanged, likely because we did not include personalized tools such as activity trackers or weekly feedback—consistent with longitudinal studies highlighting exercise adherence as a persistent challenge. Future studies should integrate objective activity monitoring to bridge this gap.

### Clinical Outcome

Our study data revealed that enrolled community-dwelling patients with hypertension exhibited well-controlled blood pressure and other cardiovascular risk factors. This observation suggests potential associations with the socioeconomic characteristics of the study population: all participants were recruited from Chengdu’s first-tier economic zone, where robust primary care management systems and higher resident health literacy may be associated with optimized baseline risk factor control. Consequently, the intervention implemented in this study demonstrated limited potential for further improvement in clinical outcomes given that the population’s preexisting optimal management status may have created a ceiling effect for measurable therapeutic efficacy. Previous longitudinal cohort studies have indicated that clinically significant improvements in anthropometric parameters (eg, waist circumference and BMI) typically require a minimum of 6 months of sustained intervention to achieve detectable changes [17]. Additionally, each 1-cm reduction in waist circumference corresponds to a cumulative energy deficit of approximately 7700 kcal based on adipose tissue energy density estimates. Achieving such energy imbalance through lifestyle interventions typically requires ≥180 days, consistent with adipose tissue turnover kinetics [18]. Existing studies have confirmed that the cardiovascular benefits of nocturnal blood pressure dipping significantly surpass those of daytime blood pressure reduction [19]. Our study found that informational support was associated with marked improvements in nocturnal blood pressure. Although few recent studies have explored the intervention effects of social support on ambulatory blood pressure, previous research has corroborated our findings [20,21]. This aligns with previous theoretical studies on social support, potentially mediated by improved sleep quality [22], enhanced treatment adherence, and buffering of psychological stress [13,23], with informational support identified as the most significant predictor [24]. Therefore, the implementation of comprehensive informational support for patients with hypertension in community settings holds promise for reducing nocturnal blood pressure and improving cardiovascular outcomes.

Additionally, no statistically significant differences were observed between pre- and postintervention levels of clinical indicators such as blood glucose and lipids. This null effect may be attributable to the relatively brief duration of our intervention. Future research should lengthen intervention periods and increase intervention intensity to optimize effectiveness.

### Strengths and Limitations

This study investigated the effects of informational support on medication adherence, blood pressure control, adoption of healthy lifestyles, and clinical indicators among patients with hypertension. The findings demonstrate significant clinical implications: observed improvements in patient adherence, nocturnal blood pressure, and select health behaviors may collectively contribute to reduced cardiovascular risk and enhanced quality of life.

Several limitations warrant consideration. First, while the prospective design addressed temporal relationships, the absence of a control group represents a critical issue that not only complicates causal inference but also makes it impossible to avoid the influence of confounding factors such as seasons. However, it is crucial to note that this feasibility pilot study aimed to generate preliminary data essential for designing methodologically robust large-scale trials. Second, there is potential reporting bias as self-reported measures constituted portions of the dataset. Third, recruitment from resource-advantaged communities within Chengdu may limit generalizability. Participants’ elevated health literacy and physicians’ structured management protocols likely yielded response patterns not fully representative of broader populations. Future investigations should prioritize multiregional sampling to enhance external validity.

### Conclusions

In conclusion, this feasibility study indicates that delivering a structured informational support intervention in a community setting is feasible and acceptable. We observed promising preliminary changes in key outcomes such as medication adherence and nocturnal blood pressure. These findings, while preliminary, justify and inform the design of a full-scale RCT to rigorously evaluate the efficacy of this approach.

## Supplementary material

10.2196/82147 Multimedia Appendix 1 Scales used in this study.

## Abbreviations

DBP: diastolic blood pressure
HLI: healthy lifestyle index
IRB: institutional review board
RCT: randomized controlled trial
SBP: systolic blood pressure
