# Supplementary material for: Personalized Informational Support for Patients With Hypertension: Single-Arm Pretest-Posttest Study
Source: JMIR Form Res. 2026 Jan 26;10:e82147. doi: 10.2196/82147 (PMC12834448; doi:10.2196/82147)
Supplement: Multimedia Appendix 1 [file formative-v10-e82147-s001.docx]

**Table S1. Medication adherence scale.**

| Number | Question | Answer |
| --- | --- | --- |
| 1 | Have you ever forgotten to take your blood pressure medication? | Yes（1） No（0） |
| 2 | Apart from forgetting to take the medication, have you missed taking your blood pressure medication for any other reasons in the past two weeks? | Yes（1）No（0） |
| 3 | When your condition worsens, have you ever stopped or reduced the use of your blood pressure medication without informing your doctor? | Yes（1）No（0） |
| 4 | When you travel or are away from home for an extended period, have you ever forgotten to bring your blood pressure medication? | Yes（1）No（0） |
| 5 | Did you take your blood pressure medication yesterday? | Yes（0）No（1） |
| 6 | If you feel that your blood pressure has decreased or is well controlled, do you plan to reduce or stop taking your blood pressure medication? | Yes（1）No（0） |
| 7 | Do you find it difficult to follow your current hypertension treatment plan? | Yes（1）No（0） |
| 8 | Do you find it difficult to remember all of your current blood pressure medications? | |
|  | No difficult（1）A little difficult（0.75）Difficult（0.5）Quite difficult（0.25）Very difficult（0） | |
| “No difficult” refers to being able to remember the medication plan all the time. “A little difficult” refers to occasionally forgetting the medication plan. “Difficult” refers to forgetting the medication plan half of the time. “Quite difficult” refers to forgetting the medication plan most of the time. “Very difficult” refers to never being able to remember the medication plan. | | |

**Table S2. Healthy lifestyle index.**

| Dimension**​** | Weight (Points)**​** | Binary Criteria (Meet Standard = Full Weight Points; Not Meet = 0 Points)**​** |
| --- | --- | --- |
| 1. Smoking​ | 2 | Meet: Non-smoker (including those who quit ≥1 year ago). Not Meet: Current smoker (including occasional/frequent smoking, ≥1 time/week). |
| 2. BMI | 1.5 | Meet: BMI 18.5–23.9 kg/m² (normal range). Not Meet: BMI <18.5 (underweight), 24–27.9 (overweight), or ≥28 (obesity). |
| 3. Physical Activity​ | 1.5 | Meet: ≥150 minutes/week of moderate-intensity exercise (e.g., brisk walking, cycling, swimming). Not Meet: <150 minutes/week or sedentary (daily steps <5,000). |
| 4. High Sodium Diet | 1 | Meet: Daily salt intake ≤6g (measured with salt-restricted spoon, including processed foods). Not Meet: Daily salt intake >6g. |
| 5. Alcohol Consumption​ | 1 | Meet: Non-drinker, or moderate drinking (male ≤25g alcohol/day, female ≤15g alcohol/day). Not Meet: Excessive drinking (male >25g/day, female >15g/day, or ≥3 binge episodes/week). |
| 6. Fruit Intake | 0.5 | Meet: Daily fresh fruit intake 200-350g (~1 apple + 1 orange, excluding juice). Not Meet: Other intakes. |
| 7. Sleep​ | 0.5 | Meet: 7–9 hours/day with good quality (fall asleep <30 mins, ≤1 awakening/night). Not Meet: <7 hours or >9 hours/day, or frequent insomnia (≥3 times/week). |
| 8. Whole Grain Intake​ | 0.5 | Meet: Whole grains account for ≥50% of staple foods. Not Meet: <50%. |
| 9. Sugar-Sweetened Beverage Restriction​ | 0.5 | Meet: Consume sugar-sweetened beverages (soda, juice drinks with added sugar) ≤1 time/week.  Not Meet: >1 time/week. |
